# Erythropoietin levels in aqueous humor of patients with glaucoma

**Published:** 2012-07-18

**Authors:** Nader Nassiri, Nariman Nassiri, Mercede Majdi, Hadi Z Mehrjardi, Yadollah Shakiba, Maryam Haghnegahdar, Amir Behzad Heidari, Ali R. Djalilian, Mahroo Mirahmadian

**Affiliations:** 1 Department of Ophthalmology, Imam Hossein Medical Center, Shahid Beheshti University of Medical Sciences, Tehran, Iran; 2 Vanak Eye Surgery Center, Tehran, Iran; and Glaucoma Division, Jules Stein Eye Institute, David Geffen School of Medicine, University of California at Los Angeles, Los Angeles, CA; 3 Department of Ophthalmology and Visual Sciences, University of Illinois at Chicago, Chicago, IL; 4 Students’ Scientific Research Center, Tehran University of Medical Sciences, Tehran, Iran; 5 Department of Immunology, School of Medicine, Tehran University of Medical Sciences, Tehran, Iran; 6 Vanak Eye Surgery Center, Tehran, Iran

## Abstract

**Purpose:**

To compare the aqueous concentration of erythropoietin (EPO) in eyes with primary open-angle (POAG), pseudoexfoliative (PXFG), and neovascular (NVG) glaucoma with age-matched eyes with cataracts, and to correlate its concentration with other factors including age, gender, intraocular pressure (IOP), type of glaucoma, and severity of glaucoma.

**Methods:**

In this prospective non-randomized comparative study, a total of 26 eyes with cataracts (control group) and 92 glaucomatous eyes (POAG, 40 eyes; PXFG, 26 eyes; NVG, 26 eyes) were enrolled. Aqueous samples (0.1 to 0.2 ml) were obtained during phacoemulsification, trabeculectomy, phacotrabeculectomy, or Ahmed valve glaucoma implants. The aqueous concentration of EPO was measured using an enzyme-linked immunosorbent assay.

**Results:**

The mean±SEM aqueous level of EPO was statistically significantly higher in eyes with glaucoma (56.7±9.3 mIU/ml) compared to the control group (0.8±0.51 mIU/ml; p<0.001). Eyes with NVG had the highest aqueous level of EPO. Aqueous EPO concentrations remained considerably elevated even in eyes with controlled IOP in all three types of glaucoma. Eyes with PXFG displayed the greatest change in aqueous EPO concentration proportionate to the IOP level. In simple regression analysis, IOP, mean deviation, and the type of glaucoma were the factors that had a statistically significantly positive correlation with the aqueous level of EPO (p=0.011 and <0.001, respectively). Only the type of glaucoma remained statistically significant in the multiple regression analysis (adjusted R^2^=0.278).

**Conclusions:**

Compared to the control group, the aqueous humor EPO concentration is increased in eyes with POAG, PXFG, and NVG, both with and without controlled IOP. The aqueous level of EPO was more proportionate to the level of IOP in eyes with PXFG compared to eyes with POAG and NVG.

## Introduction

Erythropoietin (EPO), as a 30.4 kDa (kD) glycoprotein, is an ischemia-induced paracrine factor known to be critical for the formation of red blood cells. EPO enhances red blood cell proliferation and differentiation, and also prevents apoptotic death of erythropoietin-responsive erythroid precursor cells [1,2]. In addition, both the angiogenic [3-7] and the neuroprotective [8-12] properties of EPO have been documented in several studies. EPO expression is increased in patients with glaucoma [13]. Several studies have shown that EPO concentration is increased significantly in the aqueous humor of eyes with primary open-angle glaucoma compared to eyes with only cataracts (controls) [14-16].

In this study, we compared the aqueous concentration of EPO in eyes with primary open angle glaucoma (POAG), pseudoexfoliative glaucoma (PXFG), and neovascular glaucoma (NVG) to the EPO concentration in the aqueous humor of age-matched control subjects with cataracts. We also assessed the association between the aqueous level of EPO and other factors, including age, gender, intraocular pressure (IOP), severity of visual field defect, and type of glaucoma.

## Methods

A prospective non-randomized comparative study was conducted from December 2010 to February 2011 at three medical centers in Tehran, Iran, namely, Imam Hussein Medical Center, Negah Eye Hospital, and Vanak Eye Surgery Center. The Ethics Committees at the Shahid Beheshti University (M.C.) and each clinical center approved the study protocol. All patients provided informed consent in accordance with the Declaration of Helsinki.

A total of 92 glaucomatous eyes (92 patients) and 26 age-matched control eyes (26 individuals) with senile cataracts and no glaucoma were enrolled in the study. All 26 eyes in the control group and 59 glaucomatous eyes with controlled IOP (defined as IOP ≤target pressure with anti-glaucoma medications) were candidates for phacoemulsification and intraocular lens implantation (IOL). The remaining 33 glaucomatous eyes with uncontrolled IOP (defined as IOP >target pressure with maximally tolerated anti-glaucoma medications) were candidates for trabeculectomy (9 out of 33 eyes), Ahmed glaucoma valve implants (20 out of 33 eyes), or phacotrabeculectomy and IOL implantation (4 out of 33 eyes). Only one eye of each patient was enrolled in the study. Glaucoma was defined as eyes with at least two consecutive, reliable abnormal visual field test results at baseline, defined as a pattern standard deviation (PSD) with p<0.05, and/or Glaucoma Hemifield Test (GHT) demonstrating “outside normal limits” were classified as glaucomatous regardless of the appearance of the optic disc.

Exclusion criteria for POAG and PXFG were: history of any laser and/or intraocular surgery (e.g., cataract and/or glaucoma surgeries); history of any other ocular (e.g., age-related macular degeneration) or systemic disorders (e.g., kidney disease, diabetes mellitus, cardiovascular disorders, polycythemia vera, anemia, immune disease); and history of systemic medications (e.g., chemotherapeutic agents, iron preparations, granulocyte colony-stimulating factor) that could influence the aqueous level of EPO. Among 26 eyes with neovascular glaucoma, 18 cases had diabetic retinopathy and the remainder had ischemic central retinal vein occlusion. This group of patients was only on diabetes and/or heart disease medication.

Pre-surgical assessment included identifying the type of glaucoma and conducting examinations using slit lamp biomicroscopy, fundoscopy, Snellen visual acuity (VA), baseline IOP, number of anti-glaucoma medications, and the Humphrey perimetry results. All examinations were performed within the last week before the procedure. The best-corrected visual acuity was measured using a Snellen chart (CP-690; Nidek Co, Ltd, Gamagori Aichi, Japan) calibrated for a 20-foot (approximately 6 m) distance by the line assignment method. All IOP measurements were performed by the same person (Nader Nassiri) with the Goldmann applanation tonometer (AT-900; Haag-Streit AG, Koniz, Switzerland) mounted on a slit lamp; if required, gonioscopy (Haag-Streit AG) was also performed. The IOP measured at the last visit before the operation (glaucoma, cataract, or combined surgery) was recorded and used in this study. A 24–2 threshold visual field was assessed by the Humphrey Field Analyzer (Allergan-Humphrey Instruments, San Leandro, CA); the data of a recent reliable visual field exam (within three months before the operation) was used in our study. Reliability of the visual field exam was based on the manufacturer’s criteria defined as a fixation loss of less than 20%, false positive and negative responses of less than 33%.

Aqueous humor samples (0.1 to 0.2 ml) were collected at the beginning of the operation through a paracentesis using a 27-gauge needle on a tuberculin syringe under an operating microscope. Samples were obtained carefully to avoid blood contamination. Aqueous samples were immediately cooled at <-80 °C and were protected from light. The concentration of EPO in the aqueous humor was measured by a double-antibody “sandwich” enzyme-linked immunosorbent assay (R&D Systems Inc. Minneapolis, MN). The EPO concentrations were determined by measuring the optical density at 450 nm on an ELx800 absorbance microplate reader (BioTek Instruments, Inc., Winooski, VT).

### Statistical analysis

Data analysis was performed by SPSS software, Version 17 (SPSS Inc., Chicago, IL). Based on a previous study [14], we needed at least 26 cases in each subgroup of glaucoma to find a statistically significant difference at an alpha level of 0.05 and a power of 0.85. The distribution normality of the aqueous level of EPO was checked by the Kolmogorov–Smirnov test (p<0.001). The Mann–Whitney U test was used to compare the aqueous level of EPO in the different glaucoma subgroups to the control group. Eyes were categorized based on controlled and uncontrolled IOP. The mean aqueous EPO concentration was compared within each IOP category by the Mann–Whitney U test. To determine factors independently associated with the aqueous level of EPO, a multivariate analysis was performed using a stepwise linear regression model of several variables. P-values less than 0.05 were considered statistically significant.

## Results

As illustrated in Table 1, patients without glaucoma (control group) were comparable with glaucoma patients in terms of age and gender (p=0.141 and p=0.263, respectively). The aqueous level of EPO (mean±SE) was significantly higher in glaucomatous eyes compared to eyes in the control group (56.7±9.4 mIU/ml versus 0.8±0.51 mIU/ml, respectively; p<0.001). The aqueous level of EPO was statistically significantly higher in eyes with NVG (134.9±22.1 mIU/ml) compared to POAG (18.4±4.5 mIU/ml), PXFG (17.5±7.9 mIU/ml), and the control eyes (all p-values <0.001). The difference was not statistically meaningful between PXFG and POAG (p=0.925), but both had significantly higher levels of EPO than non-glaucoma patients (p=0.041 and p=0.021, respectively).

**Table 1:** Baseline characteristics of the studied patients and mean concentration of EPO in aqueous humor of control and glaucomatous eyes.

** **	**Control**	**Glaucoma**				** **
**Variables**	**Senile cataract**	**POAG**	**PXFG**	**NVG**	**Total**	**p-value**
Number of eyes (%)	26	40 (43.4)	26 (28.3)	26 (28.3)	92	—
Age, mean±SD, years	66.3±11.2	60.6 (±13.5)	69.2 (±8.8)	58.3 (±14.1)	61.6 (±13.4)	0.141*
Female/Male	14/12	18/22	10/16	11/15	39/53	0.263**
IOP, mean±SD, mm Hg	12.7±1.5	20.0 (±9.2)	23.2 (±12.7)	25.3 (±12.7)	22.0 (±11.1)	<0.001*
BCVA, mean±SD, logMAR	0.56±0.43	0.41±0.3	0.52±0.41	1.61±0.78	0.48±0.41	0.043
No. of glaucoma medication, mean±SD	—	1.5±1.3	1.1±1.2	2.1±1.7	1.5±1.3	—
Mean deviation, mean±SD, dB	−0.12±0.3	−3.8±3.2	−2.3±2.1	−6.6±6.8	−4.8±5.0	<0.001*
Aqueous level of EPO, mean±SEM mIU/ml	0.8±0.51	18.4±4.5	17.5±7.9	134.9±22.1	56.7±9.3	<0.001*

* Mann–Whitney U test was used for analysis of EPO concentration. ** χ^2^; mIU/ml: milli international units; POAG: primary open angle glaucoma; NVG: neovascular glaucoma; PXFG: pseudoexfoliative glaucoma; IOP: intraocular pressure; BCVA: best-corrected visual acuity; EPO: erythropoietin.

Table 2 compares the aqueous level of EPO between glaucomatous eyes with and without controlled IOP. There was not a statistically significant difference between eyes with and without controlled IOP in terms of aqueous humor EPO concentration in each type of glaucoma separately (all p-values >0.05; see Table 2). There was a larger difference in the aqueous EPO concentration between eyes with controlled and uncontrolled IOP in the PXFG group compared to the POAG group (23.5 mIU/ml in the PXFG group versus 5.6 mIU/ml in the POAG group; Table 2). It seems that the aqueous EPO concentration in the PXFG group was more proportionate to the IOP level compared to the POAG group.

**Table 2:** Aqueous level of erythropoietin (mIU/ml) in the control group, and glaucomatous eyes with controlled and uncontrolled intraocular pressure.

** **	**Eyes with controlled IOP (≤target pressure)**			**Eyes with uncontrolled IOP (>target pressure)**			** **
**Groups**	**N**	**IOP (Mean±SD)**	**EPO (Mean±SE)**	**N**	**IOP (Mean±SD)**	**EPO (Mean±SE)**	p-value**
Control	26	12.6±1.5	0.8±5.4	—	—	—	—
POAG	27	14.1±3.6	14.8±6.3*	13	28.7±6.5	20.4±6.3*	0.147
PXFG	15	14.5±3.7	4.7±1.5*	11	31.5±12.2	28.2±13.9*	0.256
NVG	17	15.1±4.6	108.1±40.5*	9	32.3±11.1	168.2±39.8*	0.516

*Statistically significantly higher compared to the control group using Mann–Whitney U test (p<0.05); Target pressure: upper limit of intraocular pressure that prevents further glaucomatous damage; **Mann–Whitney U test was used for analysis, as the aqueous level of erythropoietin was not normally distributed. IOP: intraocular pressure; EPO: erythropoietin; POAG: primary open angle glaucoma; NVG: neovascular glaucoma; PXFG: pseudoexfoliative glaucoma.

Simple regression analysis showed that the level of IOP, mean deviation, and type of glaucoma had a significant correlation with the aqueous concentration of EPO (Table 3; p=0.011 and p<0.001, respectively). In the multiple regression analysis, only type of glaucoma remained statistically significant (p<0.001, adjusted R^2^=0.278; see Table 3).

**Table 3:** Determinants of erythropoietin level in aqueous humor of glaucoma patients in simple and multiple regression analyses.

** **	**Simple regression**		Multiple regression*	
**Factors**	**Magnitude (mIU/ml)**	**p-value**	**Coefficient**	**p-value**
Age, years	−0.05	0.61	−0.024	0.776
Gender	−0.14	0.12	−0.009	0.914
Intraocular pressure, mmHg	0.24	0.01	−0.049	0.538
Mean deviation, dB	−0.32	0.01	−0.073	0.348
Type of glaucoma	0.551	< 0.001	0.535	< 0.001

mIU/ml: milli international units; *Independent variables associated with the outcome at p<0.2 or having high clinical relevance were eligible for inclusion in the model. Adjusted *R^2^* for multiple regression=0.278.

## Discussion

In this prospective non-randomized comparative study, we report that the aqueous level of EPO is statistically significantly higher in eyes with POAG, PXFG, and NVG compared to age-matched eyes with only cataracts. Aqueous EPO levels remain significantly high even after reducing the IOP. Chumurch et al. [14] and Mokbel et al. [15] similarly report that the aqueous level of EPO is higher in patients with POAG compared to control patients; however, they did not detect any statistically significant difference in EPO concentrations in aqueous humor and serum. Chumurch et al. [14] did not mention the IOP level in their study population.

In the study by Mokbel et al. [15], IOP is reported as 20.6±5.1, and 19.5±6.1 in the POAG and the control groups, retrospectively. In contrast to our results, Doğu et al. [16] found that there was no statistically significant difference between eyes with PXFG and the control group with regard to the level of EPO in both humor and serum; however, they did not report IOP levels in their study population.

We found that the PXFG group had the greatest change in aqueous concentration of EPO in proportion to the level of IOP. Wang et al. [17] report that, compared to the control group with cataracts and a mean IOP of 12.0±0.8, the aqueous concentration of EPO in eyes with POAG, primary chronic angle-closure, primary acute angle-closure, and NVG were statistically significantly higher with mean±SD IOP of 25.9±1.0, 32.0±1.9, 30.7±2.1, and 36.8±6.3, respectively.

Apoptosis of the retinal ganglion cells due to elevated intraocular pressure [18] as well as hypoperfusion-induced ischemia due to disturbed retinal microcirculation [19,20] have been shown to be pathogenic mechanisms of visual deficit in glaucomatous eyes. The ischemic condition disturbs the balance between angiogenic and anti-angiogenic factors. These hypoxia-dependent events in cells appear to share a common denominator: hypoxia-inducible factor (HIF), which is a heterodimeric transcription protein [21]. It has been shown that hypoxic conditions induce the expression of the HIF-1α subunit and its target genes (including EPO) in most cells [22], both in vivo and in vitro [23]. Oxygen plays a key role in stabilizing HIF-1α and its function.

Additionally, EPO may increase secondary to an increase in glutamate, nitric oxide, and free radicals after glaucomatous damage [24]. As mentioned earlier, several studies have documented the neuroprotective role of EPO in retinal cells [8,10-12,25]. In fact, EPO is an endogenous cytokine with anti-apoptotic, anti-inflammatory, and neurotrophic properties [26]. Some experimental studies have shown exogenous EPO protected retinal ganglion cells in glaucomatous eyes through an anti-apoptosis mechanism [27].

In conclusion, we found that, compared to the eyes with cataracts in the control group, the aqueous level of EPO is significantly higher in POAG, PXFG, and NVG eyes both with and without controlled IOP. Eyes with PXFG had aqueous EPO concentrations more proportionate to IOP levels compared to eyes with POAG. However, this study has the limitation that our findings can only be linked to one population. The role of EPO in the pathophysiology of glaucoma needs to be investigated further.
